# Treatment of Diphtheria

**Published:** 1889-12-14

**Authors:** 


### TREATMENT OF DIPHTHERIA.

In a recent (October) number of the *Medical and Surgical Reporter* of Philadelphia, Dr. Arthur E. Spohn has some remarks on the use of turpentine in affections of the throat and lungs. He uses the ordinary hand atomizer, and throws a spray of pure oil of turpentine into the throat every few minutes, or at longer intervals according to the gravity of the case. The bulb of the instrument should be compressed as the act of inspiration commences, so as to ensure the application of the remedy to the whole surface. The author states "it is surprising how a diphtheritic membrane will melt away under an almost constant spray of pure oil of turpentine"; and he now uses the turpentine spray whenever a child complains of sore throat. In cases of tuberculosis of the lungs, and pneumonia, he has found turpentine inhalation very beneficial.

Mention of diphtheria reminds us that one of the recent methods of treatment advocated is by acid sublimate solution. Dr. Rennert, of Frankfort, recommends acidulating sublimate solution with tartaric acid. The formula is "bichloride of mercury one part, tartaric acid five parts, water one thousand parts." In applying the solution a wad of absorbent cotton, moistened with it, is held in a pair of forceps, and with this brush the false membrane adherent to the throat or tonsils is wiped off from below upwards. It is stated that under this treatment 62 patients recovered. It is known that corrosive sublimate is a germicide and disinfectant, and it is advanced that the addition of the acid renders it more penetrating. But there would appear to be objections to its use in the manner proposed by Rennert. Young children struggle so much under any manipulation of the kind that dangerous exhaustion may result. And as Dr. Charles W. Dulles remarks, the method is not applicable to cases in which the membrane is so extensive that it cannot all be removed, as any left would serve as a local focus of fresh growth. There is, however, great difference of opinion whether or not benefit ever results from removing the diphtheric membrane, and certainly not from scraping it away, as advised by certain continental practitioners. It is questionable if success in the treatment of diphtheria does not depend more on the condition and age of the patients, and on the hygienic conditions under which they are placed, than in the use of any medicine. Most, however, agree that warmth, good ventilation, supporting the patients' strength, and moistening the air of the sick chamber are essential. In The Hospital for January 5th, 1889, we called attention to a method of treatment very strongly advocated by Dr. Murray Gibbs, of Taranaki, New Zealand, who reported great success by the use of blue gum leaf steam (eucalyptus globulus). The author's theory is that an antiseptic agent is transmitted in the steam from the blue gum leaves, which destroys the microbes on which the malady depends. We have not, however, yet noticed corroborative evidence of the value of this method of treatment. It was then observed that the throat affection which one physician would dignify by the name of diphtheria, another physician might designate by a more homely title. Diphtheria differs much in intensity from the mildest form, which resembles an ordinary catarrhal sore throat, to the most severe character, when the malady assumes a gangrenous or putrid form. This variability of the affection leads to very different reported results in the practice of physicians—those who consider all cases of throat disease with the least exudation to be diphtheria being apparently more successful in their treatment than others who do not apply the term so generally. A reported cure, as mentioned above, of sixty-two cases leads to the suspicion that Dr. Rennert is liberal in his use of the term. Nevertheless, we think that Dr. Spohn's treatment by turpentine spray is worthy of extended trial.

